# Factors influencing the level of insight and treatment attitude: a cross-sectional study of 141 elderly patients of major depression in Guangzhou, China

**DOI:** 10.3389/fpsyt.2024.1284559

**Published:** 2024-06-05

**Authors:** Hang Yang, Jiao Chen, Junrong Ye, Tingwei Zhou, Wen Wang, Yuanxin Pan, Yanheng Wei, Xueling Lu, Lexin Yuan, Shengwei Wu, Jianxiong Guo, Aixiang Xiao

**Affiliations:** ^1^ Department of Geriatric Neurology, Affiliated Brain Hospital of Guangzhou Medical University, Guangzhou, China; ^2^ School of Nursing, Guangzhou Medical University, Guangzhou, China; ^3^ Department of Nursing Administration, Affiliated Brain Hospital of Guangzhou Medical University, Guangzhou, China; ^4^ Department of Traditional Chinese Medicine, Affiliated Brain Hospital of Guangzhou Medical University, Guangzhou, China; ^5^ Department of Chronic Diseases, Affiliated Brain Hospital of Guangzhou Medical University, Guangzhou, China

**Keywords:** depressive disorder, elderly patients, insight, ITAQ, cross-sectional study

## Abstract

**Objective:**

To explore the insight, treatment attitude, and related influencing factors of hospitalized elderly patients suffering from major depression.

**Methods:**

A total of 141 hospitalized elderly patients with depression were selected as the research objects. Insight was evaluated by the total score of the Insight and Treatment Attitude questionnaire (ITAQ). The data collected included sociodemographic characteristics, psychiatric symptoms, delirium status, social functioning, social support, suicide risk, and cognitive function.

**Results:**

The sample included 74.5% of female patients, and the mean age was 67.53 (sd=7.19) years. The influencing factors of inpatients with depression included alcohol consumption, length of hospitalization, admission types, and the main caregivers (*P*<0.05). The various factors were further analyzed by linear regression, revealing that the insight and treatment attitude of elderly depressed hospitalized patients were mainly related to the Mini-Mental State Examination (MMSE) (*β*= 0.225, *95% CI* 0.055–0.395, *P*=0.01), dependent on a caregiver (*β*=-5.810, *95% CI* -8.086~-3.535, *P*<0.001), the type of admission (involuntary admission) (*β=*-3.365, *95% CI* -5.448~-1.283, *P*=0.002), Functional Activities Questionnaire (FAQ) (*β=*-0.156, *95% CI* -0.303~-0.010, *P*=0.037), and length of stay (≤28 days) (*β=*2.272, *95% CI* 0.055~-4.489, *P*=0.045).

**Conclusion:**

The level of insight was affected by cognitive function, involuntary admission, dependent on a caregiver, social function and length of stay. Future studies should focus on cognitive function recovery, observation of admission mode, and self-care ability in elderly patients with depression.

## 1 Introduction

With the aging population, the psychological well-being of the elderly has attracted attention, with the global prevalence of depression among people over 65 years old ranges from 10% to 15% (1). Depression was regarded as one of the most common mental disorders in later life (2), therefore caring for the depressive elderly was a significant issue worldwide (3). Geriatric depression receives particular attention because it causes individuals various somatic symptoms, chronic diseases, progressive sensory impairment, decreased immune function, social isolation, low medicine adherence, and even suicidal ideation (4–7). Noticeably, researches proved the complications mentioned above were also the risk factors of deteriorating depressive symptom in aged people, which lead to a negative impact on mental health and quality of life in return (5). In 2019, a study reported that the DALYs (disability-adjusted life years) of depression among people aged 50–74 were nearly 5.2 times that of those aged 10–24, and 2.0 times that of those aged 25–49 (8). In China, the number of elderly population is 250 million, accounting for 17.9% of the total population (9). The prevalence of depression among the elderly population in China has exhibited a rising trajectory, with rates ranging from 15.9% to 23.6%, indicating a significant public health concern (10).

Insight is a good prognostic factor in the evolution and recurrence of disease. A study reported that poor self-knowledge was an important factor for low medication compliance in depressed patients, with 36.8% of depressed patients showing insufficient understanding of their disease (11). Improving insight and treatment attitude is crucial for elderly depressed patients. Therefore, insight is a significant predictor of a wide range of adverse effects. Reducing the impact of impaired insight on disease outcomes and prognosis may assist in developing accurate and clinically meaningful interventions (12). Empirical findings showed patients with mental diseases might undergo various degrees of insight impairment, previous studies pointed the prevalence of insight impairment were 60% in schizophrenia, 15 to 36% in obsessive-compulsive disorder (13, 14). A cross sectional study by He H et al. (2018) investigated patients with major depressive disorder (n=55) and bipolar depressive disorder (n=85), and found that a negative correlation between change in clinical symptoms and insight, indicating greater reduction in symptoms was associated with improved insight (15).

Understanding the risk factors of insight impairment helps to be aware of the severity of psychiatric problems in elderly patients with major depression (15). However, few studies explored the insight and treatment attitude in elderly patients with major depression. Moreover, results from longitudinal studies suggest that insight might be associated with depressive symptom (16). Therefore, identifying the factors affecting insight and treatment attitude would be beneficial for overcoming obstacles in the treatment and prognosis of elderly depression. Proposed study aimed to explore the relationship between insight and clinical characteristics of elderly patients with depression to improve prevention and treatment methods and provide more effective intervention measures.

## 2 Materials and methods

### 2.1 Subjects

A consecutive sampling method was used to recruit a total of 141 elderly patients with depression in our hospital from February 2021 to December 2023. Inclusion criteria: 1) age ≥ 55 years old; 2) the diagnosis complied with the ICD-10 criteria for depression; 3) the patient was able to respond naturally and complete scale rating; 4) the patient agreed to sign informed consent. This study had been approved by the Ethics Committee of the Affiliated Brain Hospital of Guangzhou Medical University. Informed consent was obtained from all participants or their legal guardians, who had been provided with a full explanation of the purpose and procedure of the study.

### 2.2 Questionnaire

#### 2.2.1 General information

Gender, age, educational level, marital status, smoking, alcohol consumption, main caregiver, and length of hospital stay were collected using a self-designed questionnaire.

#### 2.2.2 Brief Psychiatric Rating Scale

The BPRS, developed by Overall and Gorham (1962), was used to assess changes in psychiatric symptoms. Depending on the version of the scale, a total of 18 symptoms are included and each symptom is rated on a scale of one to seven points (17). The total score of the scale ranges between 18~126 points, with a positive correlation between the scale score and the severity of psychiatric symptoms.

#### 2.2.3 Confusion Assessment Method

The CAM, a diagnostic tool for scanning delirium with >90% sensitivity, was initially developed by Inouye et al. in 1990. It consists of nine operationalized criteria from the Diagnostic and Statistical Manual of Mental Disorders and is widely used in the assessment of delirium in a variety of patients. A score below 19 indicates that the patient does not suffer from delirium; a score between 20 and 22 indicates suspicion of delirium; a score greater than 22 indicates that the patient has delirium (18).

#### 2.2.4 Functional Activities Questionnaire

The FAQ was developed by Pfeffer et al. in 1982. The scale consists of ten items that mainly assess Instrumental Activities of Daily Living (IADL). A score below 5 indicates that the patient retains normal social functioning; a score greater than 5 indicates that the patient is unlikely to be able to live independently (19).

#### 2.2.5 Social Support Rating Scale

The SSRS was used to evaluate the level of social support of individuals. The scale contains a total of 10 items across 3 dimensions, including objective support (3 items), subjective support (4 items), and utilization of social support (3 items). The total score on the scale ranges between 10~40. The higher the overall score indicated, the greater the level of social support (20).

#### 2.2.6 The Nurses’ Global Assessment of Suicide Risk

The NGASR is an effective assessment scale suitable for nursing staff to assess the suicide risk of inpatients in the psychiatric department (21). The scale consists of 15 items, with a total score ranging from 0 to 25. Higher overall scores indicate a greater level of suicide; NGASR ≥6 indicates that the patient is at risk of suicide (22).

#### 2.2.7 Mini-Mental State Examination

Compiled by Folstein et al., the MMSE scale assesses time orientation, place orientation, immediate memory, attention and calculation, delayed memory, language, and visual space. The total score is 30 points. Higher scores indicate better cognitive function. A score below 9 indicates severe dementia; a score between 10 and 20 indicates moderate dementia; a score of 21 to 26 indicates mild dementia; and a score above 27 indicates normal cognitive function (23).

#### 2.2.8 Insight and Treatment Attitudes Questionnaire

Compiled by McEvoy et al., the ITAQ consists of 11 items (0–2 points) and evaluates patients’ perceptions of whether they have a mental illness and whether they need treatment. The total score ranges from 0 to 22, with higher scores indicating greater insight (24).

### 2.3 Data collection

This study was conducted in a tertiary public psychiatric hospital. Within the first three days of admission, an interview would be conducted to evaluate participants by research assistants. The questionnaires were rated by research assistants who had completed necessary training. The research assistants explained the purpose and significance of this study before obtaining informed consent. In this study, a total of 141 questionnaires had been distributed and had been collected, indicating an effective rate of 100%.

### 2.4 Statistical analysis

SPSS 25.0 was used to process the data. Continuous data were presented as mean ± sd, while count data were expressed using frequency and percentage. The difference between the two categories was evaluated by the independent sample t-test, or one-way ANOVA for three or more categories, and the non-parametric K independent sample test for the rank variable. Variables with statistical differences in the single factor analysis were included in univariate linear regression. Taking insight and treatment attitudes as the dependent variable, multiple linear regression analysis was performed to examine factors influencing insight and treatment attitudes in elderly patients with depression. The level of significance was set at 0.05.

## 3 Results

### 3.1 Basic situation of hospitalized elderly patients with depression

Among the 141 hospitalized elderly patients with depression, 74.5% (n=105) were female, and the mean age was 67.53 (sd=7.19) years old; 14.2% (n=20) received university education, 52.5% (n=74) received secondary education, and 33.3% (n=47) received primary education or below. Negative lifestyle habits such as drinking (4.3%) and smoking (1.4%) accounted for a small proportion. 97 cases (68.8%) were involuntarily admitted to the hospital, 111 cases (78.7%) were dependent on a caregiver, 108 cases (76.6%) were length of stay ≤28 days, and 87.2% of the elderly depressed patients took antidepressants (**Table 1**).

**Table 1:** General information of participants.

Item	Frequency	Proportion (%)
Sex		
Male	36	25.5
Female	105	74.5
Age (yrs)	67.53 ± 7.19	\
Education		
Illiteracy	10	7.1
Primary School	37	26.2
Middle School	44	31.2
High School	30	21.3
College and above	20	14.2
Marital Status		
Married	112	79.4
Divorce	5	3.5
Widowed	24	17.0
Drinking		
No	135	95.7
Yes	6	4.3
Smoking		
No	139	98.6
Yes	2	1.4
Type of Admission		
Voluntary admission	44	31.2
Involuntary admission	97	68.8
Primary caregiver		
Dependent on a caregiver	111	78.7
Self-care	30	21.3
Length of stay (days)		
≤28	108	76.6
>28	33	23.4
Types of antidepressants		
0	18	12.8
1	65	46.1
≥2	58	41.1

### 3.2 Independent sample t-test and one-way ANOVA on the influence of characteristics of different hospitalized elderly patients with depression on insight and treatment attitude

According to the grouping characteristics of different variables, the independent sample t-test or one-way ANOVA was used for analysis. The results showed that the main caregiver, admission type, smoking, alcohol consumption, and length of hospital stay were statistically significant (All *P*<0.05). Furthermore, the analysis results of FAQ, SSRS, and MMSE scores among hospitalized elderly patients with depression were statistically significant (All *P*<0.05) (**Table 2**).

**Table 2:** Single-factor analysis of ITAQ scores in depressed patients with different characteristics.

Item		ITAQ	t/F	P
Sex				
	Male	10.94 ± 7.36	-0.291	0.771
	Female	11.33 ± 6.77		
Education				
	Illiteracy	11.50 ± 6.70	0.791	0.533
	Primary School	10.30 ± 6.16		
	Middle School	10.55 ± 7.58		
	High School	11.97 ± 7.22		
	College and above	13.25 ± 6.32		
Marital Status				
	Married	11.35 ± 6.86	0.165	0.848
	Divorce	10.54 ± 7.03		
	Widowed	12.00 ± 8.28		
Drinking				
	No	11.00 ± 6.90	-2.705	0.036
	Yes	16.50 ± 4.76		
Smoking				
	No	11.17 ± 6.88	-0.984	0.327
	Yes	16.00 ± 8.49		
Type of Admission				
	Voluntary admission	15.30 ± 5.87	5.116	0.000
	Involuntary admission	9.39 ± 6.55		
MECT				
	No	10.84 ± 7.05	-1.242	0.216
	Yes	12.56 ± 6.25		
Primary caregiver				
	Dependent on a caregiver	9.69 ± 6.59	-6.800	0.000
	Self-care	16.93 ± 4.72		
Length of stay (days)				
	≤28	12.37 ± 6.80	3.696	0.000
	>28	7.52 ± 5.89		
Types of antidepressants				
	0	10.00 ± 7.44	1.298	0.276
	1	10.60 ± 7.17		
	≥2	12.33 ± 6.36		
FAQ			2.550	0.001
SSRS			1.689	0.012
MMSE			1.617	0.047
Age(yrs)			0.518	0.980

### 3.3 Analysis of influencing factors on insight and treatment attitude of hospitalized elderly patients with depression

The insight and treatment attitude of hospitalized elderly patients with depression were set as dependent variables, and the above statistically significant variables were used as independent variables. Univariate linear regression analysis was performed (*P<*0.05) and the multivariate linear regression analysis model was established. The results showed that MMSE, dependent on a caregiver, involuntary admission, FAQ, and length of stay (≤28 days) all affected the insight and treatment attitude of hospitalized elderly depressed patients (*P*<0.05). Firstly, the regression analysis results revealed higher cognitive function was associated with higher insight of elderly patients with depression *(95% CI* 0.055–0.395, *P*=0.01). Significantly lower insight and treatment attitude was observed in elderly patients with cognitive impairment. Secondly, elderly patients with depression who were unable to take care of themselves demonstrated significantly lower insight (*95% CI* -8.086~-3.535, P<0.001). Thirdly, compared with voluntarily admitted elderly depressed patients, involuntarily admitted elderly depressed patients showed lower insight and treatment attitude (*95% CI* -5.448~-1.283, *P*=0.002). Elderly patients with depression who had lower social function demonstrated significantly lower insight (*95% CI* -0.303~-0.010, *P*=0.037). Finally, elderly depressed patients who were the length of stay to the hospital had lower insight and treatment attitude (*95% CI* 0.530~-4.940, *P*=0.015) (**Tables 3**, **4**).

**Table 3:** Univariate linear regression analysis of ITAQ scores in elderly patients with depression.

Item	β	*P*
Drinking		
Yes	5.500	0.056
Type of admission		
Involuntary admission	-5.904	0.000
Primary caregiver		
Dependent on a caregiver	-7.240	0.000
Length of stay (days)		
≤28	4.855	0.000
MMSE	0.386	0.000
FAQ	-0.393	0.000
SSRS	0.329	0.000

**Table 4:** Multivariate linear regression analysis of ITAQ scores in elderly patients with depression.

	Non-standardized	Coefficients	Standardized coefficients	t	P	95% CI
	*β*	Standardized Error	Beta			
Constant	15.667	3.230	-	4.85	*P*<0.001	9.279, 22.055
Primary caregiver (Dependent on a caregiver)	-5.810	1.150	-0.346	-5.051	*P*<0.001	-8.086, -3.535
Mini-Mental State Examination (MMSE)	0.225	0.086	0.196	2.623	0.01	0.055, 0.395
Type of admission (Involuntary admission)	-3.365	1.053	-0.227	-3.196	0.002	-5.448, -1.283
Functional Activities Questionnaire (FAQ)	-0.156	0.074	-0.159	-2.107	0.037	-0.303, -0.010
Length of stay (≤28 days)	2.272	1.121	0.140	2.027	0.045	0.055,4.489

## 4 Discussion

Insight has extensively been studied in schizophrenia and bipolar disorder (25). However, few studies have investigated elderly patients with depression (26). Impaired insight was related to undesirable clinical outcomes in patients with depression, including the incidence of psychiatric hospitalization, emergency room visits, and violent or suicidal behavior (27). In addition, the poor of insight might show differential relationships to separate domains of poor neurocognition, social cognition, and/or psychiatric symptoms (28). This study explored the influencing factors of insight and treatment attitude in elderly hospitalized depression patients. Our results showed that the insight and treatment attitude of elderly patients with depression were related to cognitive function, being dependent on a caregiver, involuntary admission, social function and length of stay.

Patients with mental illness were admitted voluntarily. Article 28 of the Mental Health Law of the People’s Republic of China stipulates that patients with mental illness who hurt themselves or others can be involuntarily hospitalized. A previous study reported that patients with severe depression often denied their illness and refused to be hospitalized, in contrast with patients with medical conditions (29). This was consistent with the results of our study, showing that elderly depressed patients admitted involuntarily had a lower level of insight and treatment attitude than those who were voluntarily admitted. Schuepbach et al. (2006) also pointed out that involuntarily admitted patients were more aggressive than voluntary admission patients, and showed lower level of insight and treatment compliance (30). This was consistent with the results of our study, elderly depressed patients admitted involuntarily had a lower level of insight and treatment attitude than those who were voluntarily admitted. In addition, previous studies also had shown that physical violence and self-harm committed by a patient in psychiatric wards was associated with longer involuntary admissions (31). Compared to voluntary patients, involuntary patients had higher suicide rates and lower levels of social functioning, which might raise medical disputes and would lead to poor therapeutic relationship (32, 33). Due to lack of insight and high perceived coercion, absconding patients who were involuntarily admitted and had the tendency to harm themselves (34). Therefore, our study proposed relevant hypotheses was that suicide and self-harm in patients with involuntary admission might be affected to impaired insight in elderly patients with depression. However, O’Callaghan et al. (2022) reported that low insight was associated with involuntary admission, but few studies have attempted to quantify the need for mandatory care (35). Therefore, nurses should assess the insight of admitted patients and focus on those suicide and self-harm in patients with admitted involuntarily. The mental health care system should provide alternative forms of care for patients with higher demands.

Depression exerts a negative impact on cognitive function, including cognitive impairment in attention, memory, executive, and psychomotor domains (36). Results of the meta-analysis suggest that the nuclear components of insight were the mostly correlated with depressive symptoms, namely the awareness of having a mental illness (16). A significant relationship was also found between depressive symptoms and higher cognitive flexibility, that was higher self-awareness and reduced self-certainty (37). Startup et al. (1996) considered that high cognitive ability was associated with both high and low extremes of insight (38). Furthermore, poor cognitive insight could lead to delayed detection of disease and depressive emotion. With the improvement of depression in elderly patients, the impact of negative emotion was reduced by recognizing the relationship between cognitive insight and emotion (39). A previous study has shown that higher cognitive ability was associated with higher insight, which is beneficial to patients. However, insight alone is not sufficient (40). The current research suggested that cognitive function positively influenced insight and therapeutic attitudes.

Research has shown that increased self-care efficacy was significantly associated with improved depression, and optimal care for patients with depression included ongoing self-care support to help patients gain knowledge, skills, and confidence (41). Conversely, elderly patients with depression who depended on a caregiver had lower insight and treatment attitudes. The study by Zurkovsky (2013) showed improving cognitive function was a potential approach of relieving late-life depression (42). Elderly patients with depression showed a low self-care ability, indicating poor insight, which was more likely to cause negative emotions and burden caregivers. Improved self-care knowledge and self-efficacy were associated with better health outcomes (43). According to Remmers et al., low self-care motivation and self-confidence in elderly patients with depression could be enhanced through active management of the condition by patients, leading to improved health outcomes (44). Therefore, improving the self-care ability of elderly patients with depression is of great importance.

Nevertheless, the limitations of this study should be acknowledged. Participants were recruited from a public psychiatric hospital in Guangzhou, China, which might influence the generalization of this study. Noticeably, insight and treatment attitude were related to clinical characteristics in elderly patients with depression. Poor cognitive ability led to decreased insight. Patients who were involuntarily admitted and those with low self-care ability also showed poor insight. In conclusion, the study suggested that attention should be paid to patients’ insight and treatment attitude, and the factors affecting insight should be sought from multiple perspectives. Further research is needed to accurately quantify the level of insight in depressed patients.

## Data availability statement

The raw data supporting the conclusions of this article will be made available by the authors, without undue reservation.

## Ethics statement

The studies involving human participants were reviewed and approved by the Ethics Committee of the Affiliated Brain Hospital of Guangzhou Medical University. The patients provided their written informed consent to participate in this study.

## Author contributions

HY: Formal analysis, Writing – original draft, Writing – review & editing. JC: Formal analysis, Writing – original draft, Writing – review & editing. JY: Conceptualization, Writing – original draft, Writing – review & editing. TZ: Data curation, Writing – review & editing, Writing – original draft. WW: Data curation, Writing – review & editing, Writing – original draft. YP: Data curation, Writing – review & editing, Writing – original draft. YW: Data curation, Writing – review & editing, Writing – original draft. XL: Writing – review & editing, Data curation, Writing – original draft. LY: Writing – review & editing, Formal analysis, Writing – original draft. SW: Writing – review & editing, Formal analysis, Writing – original draft. JG: Writing – original draft, Writing – review & editing, Conceptualization. AX: Writing – original draft, Writing – review & editing, Conceptualization.

## References

[1] WHO. Estimated population-based prevalence of depression. Available at: https://www.who.int/data/gho/data/indicators/indicator-details/GHO/estimated-population-based-prevalence-of-depression

[2] Schladitz K Lobner M Stein J Weyerer S Werle J Wagner M. Grief and loss in old age: Exploration of the association between grief and depression. J Affect Disord. (2021) 283:285–92. doi: 10.1016/j.jad.2021.02.008 33578340

[3] Tanaka K. Depression-linked beliefs in older adults with depression. J Clin Nurs. (2020) 29(1-2):228–39. doi: 10.1111/jocn.15081 31661583

[4] Grover S Sahoo S Chakrabarti S Avasthi A. Anxiety and somatic symptoms among elderly patients with depression. Asian J Psychiatr. (2019) 41:66–72. doi: 10.1016/j.ajp.2018.07.009 30054249

[5] De la Torre-Luque A Ayuso-Mateos JL. The course of depression in late life: a longitudinal perspective. Epidemiol Psychiatr Sci. (2020) 29:e147. doi: 10.1017/S204579602000058X 32723402 PMC7443808

[6] Kareem YA Ogualili PN Musami UB Adebayo KO Alatishe TA Uwakwe R. DEPRESSION AND MEDICATION ADHERENCE AMONG ALDER ADULTS WITH SELECTED CHRONIC MEDICAL CONDITIONS IN MAIDUGURI, NIGERIA. West Afr J Med. (2023) 40(12 Suppl 1):S23. 38064340

[7] Shoib S Islam SMS Arafat SY Hakak SA. Depression and suicidal ideation among the geriatric population of Kashmir, India. Int J Soc Psychiatry. (2021) 67(6):651–5. doi: 10.1177/0020764020968592 33100095

[8] Diseases GBD Injuries C. Global burden of 369 diseases and injuries in 204 countries and territories, 1990–2019: a systematic analysis for the Global Burden of Disease Study 2019. Lancet. (2020) 396(10258):1204–22. doi: 10.1016/S0140-6736(20)30925-9 PMC7567026 33069326

[9] Jia L Du Y Chu L Zhang Z Li F Lyu D. Prevalence, risk factors, and management of dementia and mild cognitive impairment in adults aged 60 years or older in China: a cross-sectional study. Lancet Public Health. (2020) 5(12):e661–71. doi: 10.1016/S2468-2667(20)30185-7 33271079

[10] Tang T Jiang J Tang X. Prevalence of depressive symptoms among older adults in mainland China: A systematic review and meta-analysis. J Affect Disord. (2021) 293:379–90. doi: 10.1016/j.jad.2021.06.050 34246000

[11] Yen CF Chen CC Lee Y Tang TC Ko CH Yen JY. Insight and correlates among outpatients with depressive disorders. Compr Psychiatry. (2005) 46(5):384–9. doi: 10.1016/j.comppsych.2004.11.004 16122540

[12] Xavier RM Pan W Dungan JR Keefe RSE Vorderstrasse A. Unraveling interrelationships among psychopathology symptoms, cognitive domains and insight dimensions in chronic schizophrenia. Schizophr Res. (2018) 193:83–90. doi: 10.1016/j.schres.2017.07.002 28693755

[13] Gan J He J Fu H Zhu X. Association between obsession, compulsion, depression and insight in obsessive-compulsive disorder: a meta-analysis. Nord J Psychiatry. (2022) 76(7):489–96. doi: 10.1080/08039488.2021.2013532 34895018

[14] Buckley PF Wirshing DA Bhushan P Pierre JM Resnick SA Wirshing WC. Lack of insight in schizophrenia: impact on treatment adherence. CNS Drugs. (2007) 21(2):129–41. doi: 10.2165/00023210-200721020-00004 17284095

[15] He H Chang Q Ma Y. The association of insight and change in insight with clinical symptoms in depressed inpatients. Shanghai Arch Psychiatry. (2018) 30(2):110–8. doi: 10.11919/j.issn.1002-0829.217149 PMC5936037 29736131

[16] Belvederi Murri M Respino M Innamorati M Cervetti A Calcagno P Pompili M. Is good insight associated with depression among patients with schizophrenia? Systematic review and meta-analysis. Schizophr Res. (2015) 162(1-3):234–47. doi: 10.1016/j.schres.2015.01.003 25631453

[17] Lukoff D Liberman RP Nuechterlein KH. Symptom monitoring in the rehabilitation of schizophrenic patients. Schizophr Bull. (1986) 12(4):578–602. doi: 10.1093/schbul/12.4.578 3810065

[18] Inouye SK van Dyck CH Alessi CA Balkin S Siegal AP Horwitz RI. Clarifying confusion: the confusion assessment method. A new method for detection of delirium. Ann Intern Med. (1990) 113(12):941–8. doi: 10.7326/0003-4819-113-12-941 2240918

[19] Pfeffer RI Kurosaki TT Harrah CH Jr Chance JM Filos S. Measurement of functional activities in older adults in the community. J Gerontol. (1982) 37(3):323–9. doi: 10.1093/geronj/37.3.323 7069156

[20] Xu Q Liu D Zeng F Luo H Zuo X Li Y. Social support and management strategies for chronic disease in patients with systemic lupus erythematosus. Zhong Nan Da Xue Xue Bao Yi Xue Ban. (2019) 44(1):67–73. doi: 10.11817/j.issn.1672-7347.2019.01.011 30837405

[21] Cutcliffe JR Barker P. The nurses’ Global assessment of suicide risk (NGASR) developing. J Psychiatr Ment Health Nurs. (2004) 11(4):393–400. doi: 10.1111/j.1365-2850.2003.00721.x 15255912

[22] Ferrara P D'Agostino A Destrebecq A. Predictive validity of the NGASR in suicide attempts and early readmission to a psychiatric inpatient unit. Psychiatr Serv. (2019) 70(11):1072. doi: 10.1176/appi.ps.201900185 31672108

[23] Folstein MF Folstein SE McHugh PR. "Mini-mental state". A practical method for grading the cognitive state of patients for the clinician. J Psychiatr Res. (1975) 12(3):189–98. doi: 10.1016/0022-3956(75)90026-6 1202204

[24] McEvoy JP Apperson LJ Appelbaum PS Ortlip P Brecosky J Hammill K. Insight in schizophrenia. Its relationship to acute psychopathology. J Nerv Ment Dis. (1989) 177(1):43–7. doi: 10.1097/00005053-198901000-00007 2562850

[25] Pini S Cassano G Dell'Osso L Amador XF. Insight into illness in schizophrenia, schizoaffective disorder, and mood disorders with psychotic features. Am J Psychiatry. (2001) 158(1):122–5. doi: 10.1176/appi.ajp.158.1.122 11136644

[26] Van Camp L Van Den Ameele S Sabbe BGC Oldenburg JFE. The longitudinal course of cognitive insight and mood in bipolar disorder. Psychiatry Res. (2018) 269:9–12. doi: 10.1016/j.psychres.2018.08.063 30144670

[27] Lysaker PH Vohs J Hillis JD Kukla M Popolo R Salvatore G. Poor insight into schizophrenia: contributing factors, consequences and emerging treatment approaches. Expert Rev Neurother. (2013) 13(7):785–93. doi: 10.1586/14737175.2013.811150 23898850

[28] Subotnik KL Ventura J Hellemann GS Zito MF Agee ER Nuechterlein KH. Relationship of poor insight to neurocognition, social cognition, and psychiatric symptoms in schizophrenia: A meta-analysis. Schizophr Res. (2020) 220:164–71. doi: 10.1016/j.schres.2020.03.038 32334936

[29] Hustoft K Larsen TK Bronnick K Joa I Johannessen JO Ruud T. Psychiatric patients' attitudes towards being hospitalized: a national multicentre study in Norway. BMC Psychiatry. (2022) 22(1):726. doi: 10.1186/s12888-022-04362-8 36414961 PMC9682722

[30] Schuepbach D Goetz I Boeker H Hell D. Voluntary vs. involuntary hospital admission in acute mania of bipolar disorder: results from the Swiss sample of the EMBLEM study. J Affect Disord. (2006) 90(1):57–61. doi: 10.1016/j.jad.2005.09.012 16324749

[31] Iozzino L Ferrari C Large M Nielssen O De Girolamo G. Prevalence and risk factors of violence by psychiatric acute inpatients: A systematic review and meta-analysis. PLoS One. (2015) 10(6):e0128536. doi: 10.1371/journal.pone.0128536 26061796 PMC4464653

[32] Kallert TW Glöckner M Schützwohl M. Involuntary vs. voluntary hospital admission. A systematic literature review on outcome diversity. Eur Arch Psychiatry Clin Neurosci. (2008) 258(4):195–209. doi: 10.1007/s00406-007-0777-4 18080170

[33] Sheehan KA Burns T. Perceived coercion and the therapeutic relationship: a neglected association? Psychiatr Serv. (2011) 62(5):471–6. doi: 10.1176/ps.62.5.pss6205_0471 21532071

[34] Gowda GS Thamby A Basavaraju V Nataraja R Kumar CN Math SB. Prevalence and Clinical and Coercion Characteristics of Patients who Abscond during Inpatient Care from Psychiatric Hospital. Indian J Psychol Med. (2019) 41:144–9. doi: 10.4103/IJPSYM.IJPSYM_188_18 PMC6436412 30983662

[35] O'Callaghan AK Plunkett R Kelly BD. The association between objective necessity for involuntary treatment as measured during admission, legal admission status and clinical factors in an inpatient psychiatry setting. Int J Law Psychiatry. (2022) 81:101777. doi: 10.1016/j.ijlp.2022.101777 35051849

[36] Gonda X Pompili M Serafini G Carvalho AF Rihmer Z Dome P. The role of cognitive dysfunction in the symptoms and remission from depression. Ann Gen Psychiatry. (2015) 14:27. doi: 10.1186/s12991-015-0068-9 26396586 PMC4578787

[37] Riggs SE Grant PM Perivoliotis D Beck AT. Assessment of cognitive insight: a qualitative review. Schizophr Bull. (2012) 38(2):338–50. doi: 10.1093/schbul/sbq085 PMC3283158 20693342

[38] Startup M. Insight and cognitive deficits in schizophrenia: evidence for a curvilinear relationship. Psychol Med. (1996) 26(6):1277–81. doi: 10.1017/S003329170003600X 8931174

[39] Lincoln TM Lullmann E Rief W. Correlates and long-term consequences of poor insight in patients with schizophrenia. A systematic review. Schizophr Bull. (2007) 33(6):1324–42. doi: 10.1093/schbul/sbm002 PMC2779879 17289653

[40] Cooke MA Peters ER Greenwood KE Fisher PL Kumari V Kuipers E. Insight in psychosis: influence of cognitive ability and self-esteem. Br J Psychiatry. (2007) 190:234–7. doi: 10.1192/bjp.bp.106.024653 17766764

[41] Bodenheimer T Wagner EH Grumbach K. Improving primary care for patients with chronic illness. JAMA. (2002) 288(14):1775–9. doi: 10.1001/jama.288.14.1775 12365965

[42] Zurkovsky L Taylor WD Newhouse PA. Cognition as a therapeutic target in late-life depression: potential for nicotinic therapeutics. Biochem Pharmacol. (2013) 86(8):1133–44. doi: 10.1016/j.bcp.2013.07.032 PMC3856552 23933385

[43] Ludman EJ Peterson D Katon WJ Lin EH Von Korff M Ciechanowski P. Improving confidence for self care in patients with depression and chronic illnesses. Behav Med. (2013) 39(1):1–6. doi: 10.1080/08964289.2012.708682 23398269 PMC3628828

[44] Remmers C Hibbard J Mosen DM Wagenfield M Hoye RE Jones C. Is patient activation associated with future health outcomes and healthcare utilization among patients with diabetes? J Ambul Care Manage. (2009) 32(4):320–7. doi: 10.1097/JAC.0b013e3181ba6e77 19888008

